# Assessment of spinal cord injury using ultrasound elastography in a rabbit model in vivo

**DOI:** 10.1038/s41598-023-41172-8

**Published:** 2023-09-15

**Authors:** Songyuan Tang, Bradley Weiner, Francesca Taraballi, Candice Haase, Eliana Stetco, Shail Maharshi Mehta, Peer Shajudeen, Matthew Hogan, Enrica De Rosa, Philip J. Horner, K. Jane Grande-Allen, Zhaoyue Shi, Christof Karmonik, Ennio Tasciotti, Raffaella Righetti

**Affiliations:** 1 Department of Electrical and Computer Engineering, Texas A&M University, College Station, TX USA; 2 Orthopedics and Sports Medicine, Houston Methodist Hospital, Houston, TX USA; 3 Department of Orthopedics and Sports Medicine, Center for Musculoskeletal Regeneration, Houston Methodist Hospital, Houston, TX USA; 4 Department of Bioengineering, Rice University, Houston, TX USA; 5 Department of Neurosurgery, Center for Neuroregeneration, Houston Methodist Research Institute, Houston, TX USA; 6 Translational Imaging Center, Houston Methodist Research Institute, Houston, TX USA; 7 Department of Human Sciences and Promotion of Quality of Life, San Raffaele Roma Open University and IRCCS San Raffaele Pisana, 00166 Rome, Italy

**Keywords:** Biomarkers, Medical research, Engineering

## Abstract

The effect of the mechanical micro-environment on spinal cord injury (SCI) and treatment effectiveness remains unclear. Currently, there are limited imaging methods that can directly assess the localized mechanical behavior of spinal cords in vivo. In this study, we apply new ultrasound elastography (USE) techniques to assess SCI in vivo at the site of the injury and at the time of one week post injury, in a rabbit animal model. Eleven rabbits underwent laminectomy procedures. Among them, spinal cords of five rabbits were injured during the procedure. The other six rabbits were used as control. Two neurological statuses were achieved: non-paralysis and paralysis. Ultrasound data were collected one week post-surgery and processed to compute strain ratios. Histologic analysis, mechanical testing, magnetic resonance imaging (MRI), computerized tomography and MRI diffusion tensor imaging (DTI) were performed to validate USE results. Strain ratios computed via USE were found to be significantly different in paralyzed versus non-paralyzed rabbits. The myelomalacia histologic score and spinal cord Young’s modulus evaluated in selected animals were in good qualitative agreement with USE assessment. It is feasible to use USE to assess changes in the spinal cord of the presented animal model. In the future, with more experimental data available, USE may provide new quantitative tools for improving SCI diagnosis and prognosis.

## Introduction

Traumatic spinal cord injury (SCI) is damage to the spinal cord resulting from an external physical impact; commonly as the result of a motor vehicle accident, fall, or sports-related injury^1^. The estimated acute in-hospital mortality of traumatic SCI ranges from 4 to 17%^1^. In the USA, the average lifetime cost associated with traumatic SCI is between US$ 0.5 and 2 million per patient^2^.

In traumatic SCI, the initial forces delivered to the spinal cord is known as the primary injury, while the ensuing cellular, molecular and biochemical phenomena that continue to self-destruct the spinal cord and impede neurological recovery is referred to as the secondary injury^3, 4^. SCI typically starts with hemorrhage, rapid necrosis and is followed by neuroinflammation that involves a variety of cell phenotypes^3, 5^. Several weeks post injury, cavities, scar tissues are formed and white matter undergoes different stages of degenerative processes^5^. Glial scar tissues are formed around the injury epicenter^6, 7^ and have divergent roles in neuro-regeneration^8^. As of now, the pathophysiology of SCI has not been fully elucidated^1, 9, 10^. A clearer understanding of a spine trauma’s pathophysiological sequelae can potentially benefit SCI patient care, especially diagnosis and prognosis.

Medical imaging is commonly coupled with clinical examination to guide decision making in early stages of spinal trauma. To date, the most effective imaging method to assess neural tissues is magnetic resonance imaging (MRI)^2^. In particular, T2-weighted MRI has been demonstrated to predict neurological outcomes^11^ of SCI whereas more advanced protocols leveraging diffusion, magnetization transfer, and metabolism^12–14^ have recently been shown in the research setting to produce quantitative imaging markers of SCI and to improve prognostication. Diffusion tensor imaging (DTI) has been investigated to visualize the spinal cord white matter structure^13^ by applying mathematical models^13, 15^. The development of new imaging methods of clinical viability to assess SCI could be of great clinical significance. In particular, ultrasound (US) is safe, fast, portable, relatively inexpensive, and readily available in an operative suite. In the context of SCI imaging, several groups have investigated the addition of contrast agents to US imaging to visualize and quantify the pattern of spinal cord blood flow (SCBF)^16–18^ and monitor its time course^19^.

Structurally and mechanically, the spinal cord is distinct from its surrounding tissues and possesses complex properties. In general, a traumatic injury has a direct impact on these properties as manifested in the disruption of neural axons, blood vessels and neural-cell membranes accompanying the initial mechanical trauma^1, 2^. Subsequent molecular and cellular events may profoundly alter the micromechanical environment of a spinal cord, such as the distributions of intraspinal pressure and blood pressure, thereby obstructing the blood flow and leading to injury worsening^16, 19^. Furthermore, injury-related changes in mechanical properties of the spinal cord tissue could have significant implications on neuronal regrowth^20^. More specifically, several types of cells in the nervous system, including sensory neurons, oligodendrocytes, astrocytes and microglial cells have been shown to sense and adapt to the stiffness of their environment^20–22^. For example, an earlier study on mouse spinal cord primary neuronal cells showed more than 3 times of neurite branching when cells were grown on softer substrates compared to those grown on stiffer ones^23^. Thus, availability of additional markers to detect clinically relevant mechanical changes might improve SCI diagnosis and prognosis. Recently, several studies have utilized atomic force microscopy (AFM) indentation experiments to characterize the Young’s modulus of glial scars following SCI in dedicated animal models^20, 24^. The main limitation of AFM is that measurement can only be performed on the surface of cells^25^. Although in vivo AFM imaging platforms have been developed for small animals during their embryonic development ^26^, the clinical translation of AFM to in vivo tissue stiffness assessment remains challenging. Other model-based quantitative markers such as those derived from DTI could also be useful in predicting neural tissue mechanical behaviors^27^, however, they can not directly assess the mechanical behavior of the spinal cord in vivo.

Ultrasound elastography (USE)^28^ is a quantitative imaging technique used to measure the mechanical behavior of tissues. It maintains ultrasonography’s advantages in safety, portability, non-invasiveness, with high spatial and temporal resolutions. In a typical USE experiment, a small uniaxial compression is applied to the tissue while a sequence of radio-frequency (RF) data is acquired^29^. The local distribution of the strains that is generated in the tissue due to the compression is mapped into images called “elastograms” and this specific type of elastography is called “strain elastography”. Among the various strains that can be computed from the RF data, the axial normal strain (computed in the direction of the applied compression) is typically used to assess underlying tissue stiffness changes^30^. Strain elastography has been applied to various tissues and organs such as the breast^31^, heart^32^, artery^33^ and muscle^34^. In addition to strain elastography, shear wave elastography (SWE) applies acoustic radiation forces to the tissue and measures shear wave propagation parameters. To estimate mechanical parameters such as Young’s modulus, it relies on mathematical models and prior boundary conditions^35^. In several recent studies, SWE was applied to assess elasticity of compressed and uncompressed spinal cords from animal models and human patients^36, 37^. In the field of tissue engineering, another recent study applied SWE to measure spinal cord stiffness in a canine model of myelopathy, with the intent of advancing regeneration therapies using stiffness-matched hydrogel^38^.

In this paper, we present a new method to detect SCI in an in vivo rabbit animal model with spinal cord injury exhibiting distinct neurological statuses of non-paralysis and paralysis. This method is based on the use of USE and does not rely on contrast agents or sophisticated apparatus for data acquisition. From the RF data acquired during elastography experiments, we derived the ratio of axial normal strains from the spinal cord to those from the surrounding tissue, evaluated the strain ratios in transverse imaging planes and compared the results from non-paralyzed and paralyzed rabbits in vivo. Independent histologic analysis, mechanical testing, and DTI tractography were performed on selected rabbits to validate results obtained from USE.

## Material and methods

### Animals and treatments

In vivo study reported in this paper is in accordance with ARRIVE (Animal Research: Reporting of In Vivo Experiments) guidelines. This study was approved by the Houston Methodist Research Institute Institutional Animal Care and Use Committee (IACUC) (study#: AUP-0718-0041). All experiments were performed in accordance with relevant guidelines and regulations. Eleven New Zealand White rabbits (obtained through Charles River) were used and divided into 2 groups: with injury (n = 5) and controls (n = 6). The surgery was performed by a spine surgeon. Prior to surgery, rabbits were taken to the operating room and anesthetized with isoflurane. They were then prepped and draped in sterile fashion. With the rabbit in the prone position, a dorsal skin incision of 3 cm in length was made 2 cm lateral to the midline over the L4-S1 vertebral bodies. Electrocautery was used to expose the spinous processes and laminae of L4 and L5. A midline laminectomy of L5 was performed with Kerrisons. With standard laminectomy in both the control and experimental groups, the #2 Kerrison was used to create a crush injury to the spinal cord in the experimental group. Following this, hemostasis was obtained and the wound irrigated. The wound was then closed in three layers in standard fashion. After the surgery, rabbits were returned to private cages with free food, water and ambulation allowed ad libitum. All rabbits were euthanized with isoflurane overdose approximately 7 days post-op.

### US data acquisition

US images were acquired in vivo using a 38-mm linear array transducer (Sonix RP, Ultrasonix, Richmond, BC, Canada) on the day of euthanasia. Prior to imaging, each rabbit was lying in a prone position under general anesthesia. Hair on the lower back was clipped and an acoustic transmission gel was applied. For each rabbit, the region of interest (ROI) was the spinal canal across the laminectomy defect area. The imaging protocol included US elastography and B-mode imaging. Acquisition protocols are detailed below.

#### US elastography

Elastography was performed with the transducer having a center frequency of 6.6 MHz and 5–14 MHz bandwidth, 1 mm lateral beamwidth in the focal plane (spinal canal)^39–44^. A compressor plate was attached to the transducer probe, and a compressive load was uniformly applied to the tissue. RF data were collected during compression both from axial and sagittal probe orientations.

#### B-mode US

B-mode imaging was performed at a center frequency of 10 MHz^45–48^. For each rabbit, the transducer probe was moved on the skin surface along the cranial-caudal axis from approximately L4 to S1. A video clip (cineloop data) was recorded in this meantime.

### MR data acquisition

MR images were acquired in vivo using a Siemens MAGNETOM Vida 3T system (Siemens Healthineers, Erlangen, Germany) on the day of euthanasia. An 18-channel receive knee coil (Nova Medical Inc.) was centered at the L4–L7 region of the rabbit spinal cord in the isocenter of the magnet. The imaging protocol included that of T1- and T2-weighted imaging and DTI. T2-weighted spin-echo structural images were acquired in sagittal view (repetition time (TR) = 3000 ms, echo time (TE) = 94 ms, field of view (FOV) = 90 × 104 mm^2^, spatial resolution = 0.3 × 0.3 × 1.5 mm^3^, flip angle (FA) = 150°). T1-weighted spin-echo anatomical images were acquired in axial view to locate the spinal cord (TR = 787 ms, TE = 13 ms, FOV = 100 × 40 mm^2^, spatial resolution = 0.3 × 0.3 × 2.0 mm^3^, FA = 130°). Diffusion data were acquired with a spin-echo diffusion-weighted echo planar imaging (EPI) sequence (b = 800 s/mm^2^, 30 directions, TR = 4700 ms, TE = 93 ms, FOV = 164 × 94 mm^2^, spatial resolution = 1.4 × 1.4 × 1.5 mm^3^).

### CT data acquisition

CT scans were performed by Biograph Vision (Siemens Healthcare, Erlangen, Germany) with 100 kV and 190–210 mA. The image data were reconstructed to a matrix of 512 × 512 pixels. The pixel dimension was 0.2695 × 0.2695 mm^2^ on the trans-axial plane with slice separation of 0.5 mm along z-axis (cranio-caudal).

### Mechanical testing

Spinal cords were harvested from the lower lumbar (L4-L6) spine of freshly sacrificed rabbits. Segments of spinal cord were extracted from the spinal canal by removing laminae then severing the nerve roots and dural sac to release the spinal cord. Isolated cord segments were stored in sterile 1X phosphate-buffered saline (PBS) at 4℃ and immediately brought to the mechanical testing facility. Prior to mechanical testing, each spinal cord was cut into approximately 3 mm longitudinal samples. Samples were placed in 1X PBS at 37℃ and loaded in the transverse orientation in a mechanical compression testing device (Bose Enduratec ELF 3200 system; Bose, Eden Prairie, MN). The actuator was driven until 70% strain at a strain rate corresponding to approximately 0.4/min. Engineering stress–strain curves for each sample were plotted from the results of the compression test. Young’s modulus of each sample was identified as the slope of a linear fit to the stress–strain curve between 10% and 20% strain.

### Histology

Spine samples (with surrounding muscle intact) centered at the laminectomy site were harvested and fixed in 10% neutral buffered formalin. Each sample was scanned with a North Star Imaging X50 (Rogers, Minnesota) micro-CT machine and data reconstructed using the efX-CT software. With two transverse planes representing selected ROIs during USE annotated in the CT reconstruction, the sample was sectioned and cross-sections from these designated ROIs were acquired. The cross-sections were then immersed in Formical-4 solution for decalcification, dehydrated in a graded alcohol and embedded in paraffin. Histological slides were stained with H&E and the digital images were acquired with an EVOS FL Auto microscope (Invitrogen). Semi-quantitative analysis was performed by a board-certified veterinary pathologist. Histologic scores on the disruption of architecture (malacia) of the spinal cord were evaluated in its dorsal and ventral regions, respectively.

### Data post-processing

#### US elastography

Volumetric CT, B-mode US, T1- and T2-weighted MR images were co-registered at the site of laminectomy using a dedicated software^49^. RF data acquired during USE were then located in this laminectomy defect area by matching anatomic landmarks in their representative envelop-detected image and the volumetric B-mode image. The RF data were processed using a strain estimation algorithm^50^. The ratio of the mean strain in the spinal cord to that in the soft tissue was computed and repeated measures were averaged. For each animal, strain ratios from distinct neighborhoods across the laminectomy site were further averaged and final results were reported as mean ± standard deviation (SD) over all non-paralyzed rabbits and paralyzed rabbits.

#### DTI tractography

DTI sequences were uploaded into DSI Studio for fiber reconstruction. A mask was applied to optimally segment the spinal cord from background adjusting threshold, smoothing and considering anatomy in co-registered CT and T2-weighted MR images. Reconstruction was performed using the generalized Q-sampling imaging (GQI) method^51^ followed by fiber tracking in the resulting quantitative anisotropy (QA) map^52^.

### Statistical analysis

Statistical analysis was performed using Graphpad Prism software. Two-tailed Wilcoxon rank sum test was performed on the mean strain ratio of each animal (α = 5%).

## Results

### Assessment of SCI using USE

**Figure 1:**
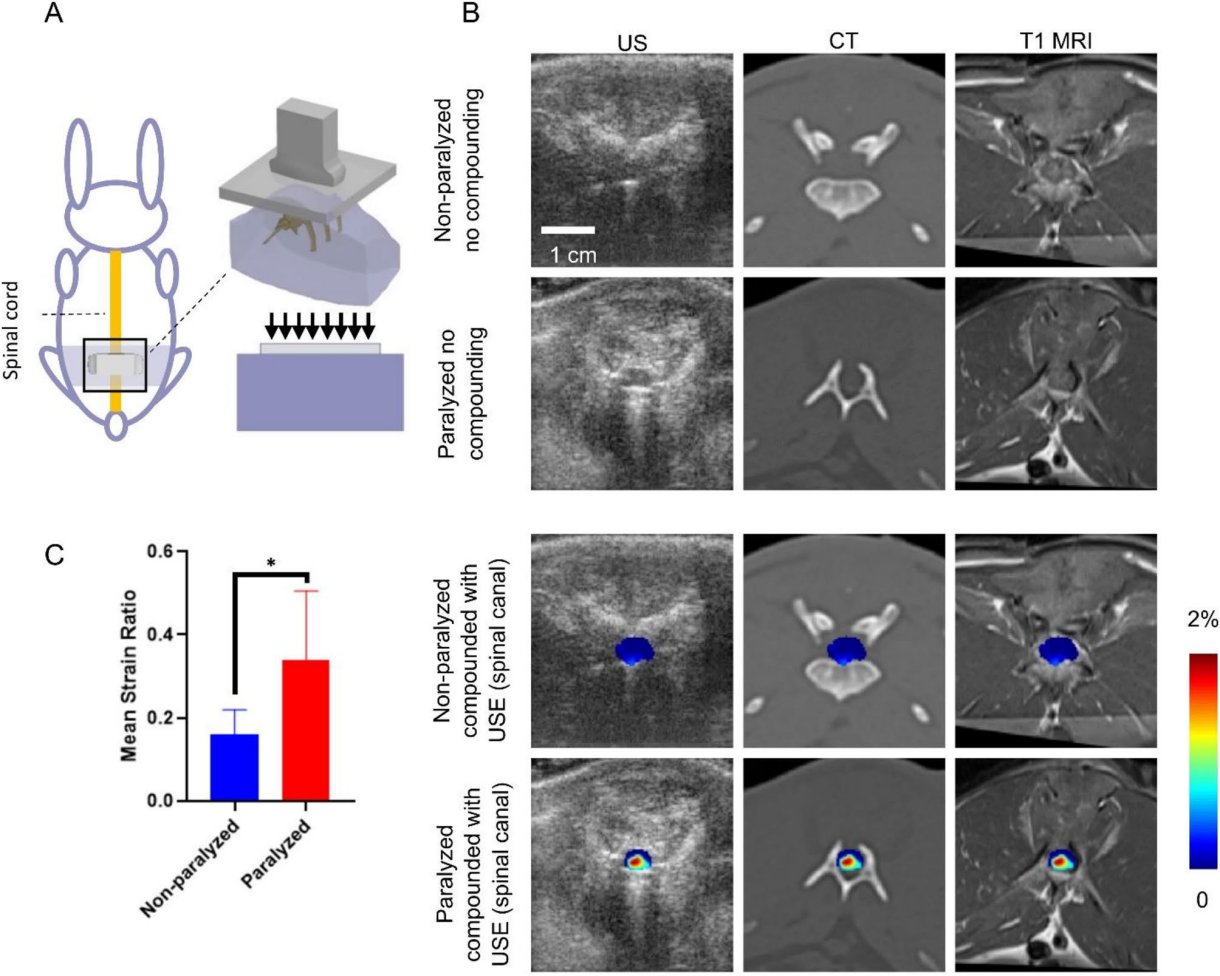
Comparison of multimodal imaging results in representative transverse planes and summary of quantitative results obtained from USE. (**A**) Schematic of the rabbit animal model and USE imaging of the spinal cord in vivo: relative position of the scan in the animal’s dorsal view and in 3-D. The expected compression field is uniform when sufficient contact is ensured (transducer not shown and tissue approximated as a cuboid for clarity of illustration). (**B**) B-mode image (column 1), CT image (column 2) and T1-weighted MR image (column 3) alone (rows 1–2) and compounded with axial normal strain elastogram (spinal canal segmented, rows 3–4) from a non-paralyzed rabbit (rows 1 and 3) and a paralyzed rabbit (rows 2 and 4). (**C**) Mean strain ratios computed from non-paralyzed rabbits (n = 6) and paralyzed rabbits (n = 5); *p < 0.05.

Eleven (6 non-paralyzed and 5 paralyzed) rabbits were evaluated using USE (Fig. 1A). B-mode US, CT and T1-weighted MR images alone and compounded with axial normal strain elastograms (spinal canal segmented and normalized with respect to the surrounding soft tissues) acquired from representative transverse planes of a non-paralyzed rabbit and a paralyzed rabbit are presented (Fig. 1B). It can be seen that differences in axial normal strains derived from USE of the non-paralyzed rabbit and paralyzed rabbit are clearly evident. For the non-paralyzed rabbit, the strains are low in magnitude and relatively uniform across the cross section of the spinal cord, whereas, for the paralyzed rabbit, the strains exhibit higher spatial variation, and a considerable region of the tissue exhibit significantly higher strains. Statistical analysis (Fig. 1C) shows significantly higher mean USE strain ratios from the group of paralyzed rabbits (0.34 ± 0.17) than non-paralyzed rabbits (0.16 ± 0.06, *p* < 0.05).

### Association between USE and histology

**Figure 2:**
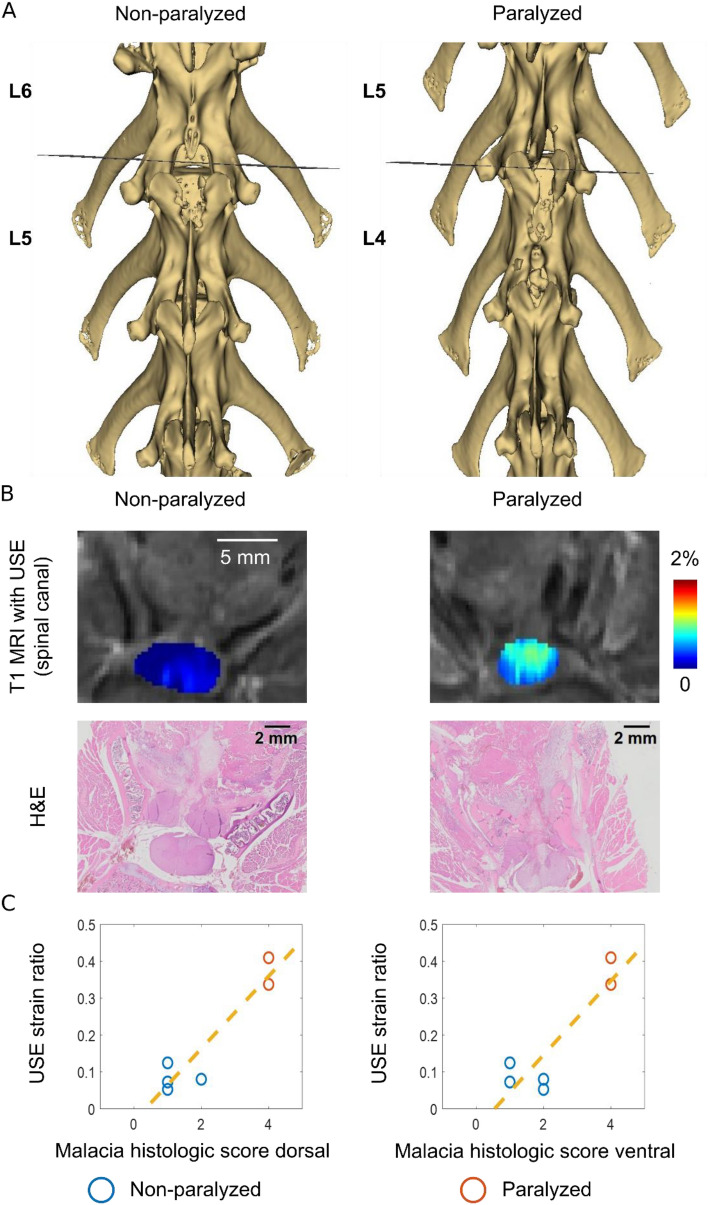
Association between USE and histologic analysis. (**A**) Axial planes to be analyzed referenced to the vertebral bone model reconstructed from CT. B: T1-weighted MR images compounded with axial normal strain elastograms (spinal canal segmented, row 1) acquired from the designated planes and H&E histological sections (row 2) acquired from the designated planes. In (**A**, **B**), the left column corresponds to a non-paralyzed rabbit and the right column corresponds to a paralyzed rabbit. (**C**) Scatter plots of the strain ratio measured by USE and semi-quantitative histologic score of malacia. The malacia score was evaluated in the dorsal and ventral areas of the spinal cords.

Two spine specimens originally from non-paralyzed rabbits and another specimen from a paralyzed rabbit were harvested for histologic analysis. From each specimen, two planes that matched the USE imaging planes were identified and histologically processed. Representative transverse planes of a non-paralyzed rabbit and a paralyzed rabbit are presented (Fig. 2A). In each plane, T1-weighted MR images compounded with axial normal strain elastograms (spinal canal segmented and normalized with respect to the surrounding soft tissues) and their coplanar hematoxylin and eosin-stained (H&E) histological images are compared (Fig. 2B). For the non-paralyzed rabbit, strains from USE are uniformly low across the spinal cord cross section (Fig. 2B, column 1, row 1). From histology, the spinal cord’s white matter is mildly to markedly diffusely vacuolated. Scattered neuronal cell bodies within the gray matter of the spinal cord show degenerative changes ranging from central chromatolysis to disruption of neuronal content and cytoplasmic borders (Figs. 1 and 2 of the supplementary information). For the paralyzed rabbit, strains from USE are more pronounced across the spinal cord cross sections (Fig. 2B, column 2, row 1). From histology, the spinal cord’s white matter is markedly, diffusely vacuolated and an extensive dorsal region of white matter is severely disrupted with near-obliteration of normal architecture and extrusion of degenerate white matter into tissues immediately dorsal to the spinal canal (Fig. 3 of the supplementary information). The gray matter of the spinal cord exhibits marked diffuse degenerative changes with hemorrhage, disruption of neuronal cytoplasmic borders and accumulation of macrophages and microglial cells (Fig. 4 of the supplementary information).

From the six designated planes, malacia histologic scores were plotted with the corresponding USE strain ratio. A linear regression line was overlaid on each scatter plot (Fig. 2C).

### Association between USE and mechanical testing

**Figure 3:**
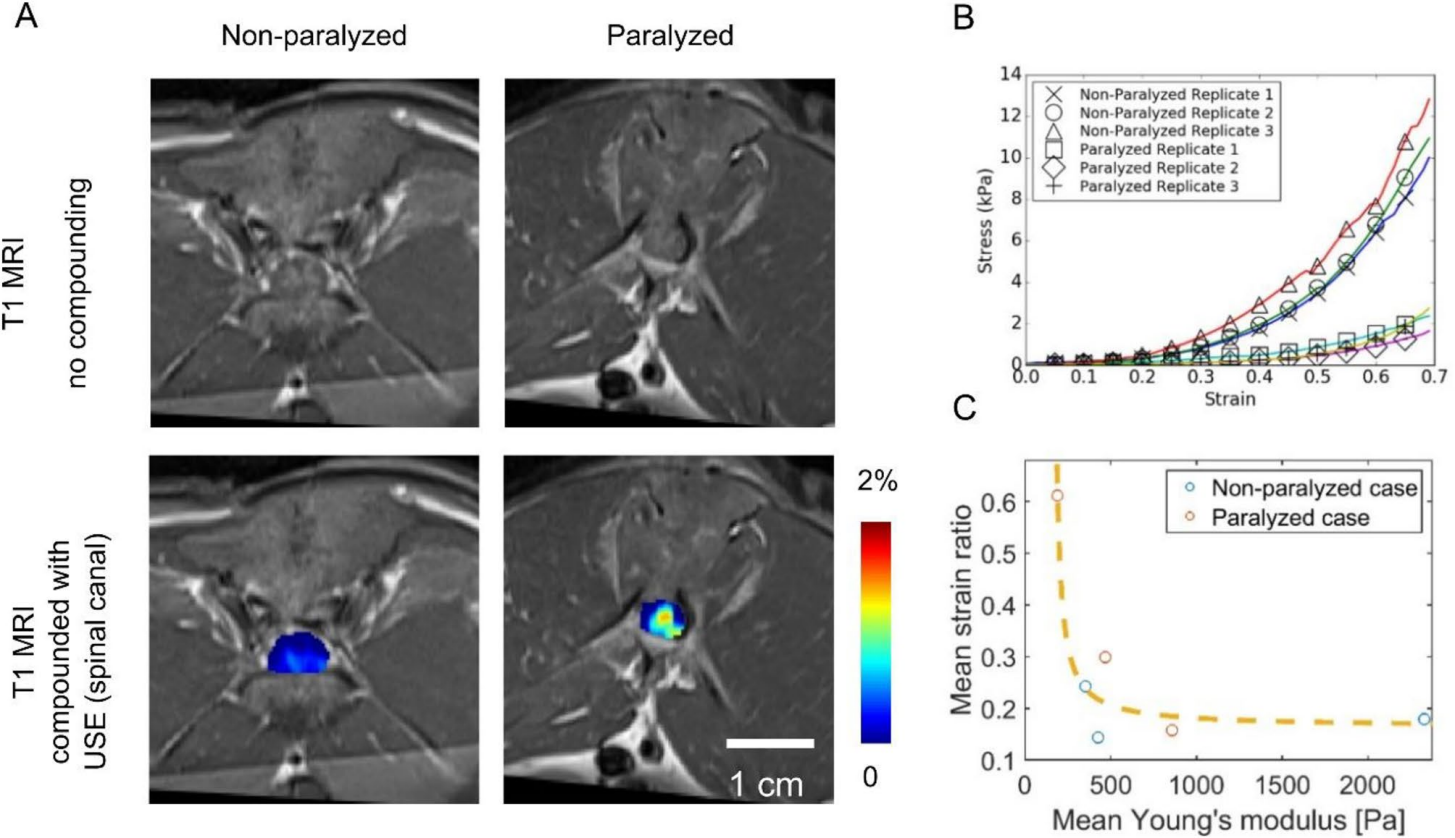
Association between USE and mechanical testing. (**A**) T1-weighted MR images alone (top) and compounded with axial normal strain elastogram (bottom, with spinal canal segmented) from a non-paralyzed rabbit (left) and a paralyzed rabbit (right). (**B**) Transverse plane engineering stress–strain curves of spinal cords extracted from the same rabbits as in (**A**). (**C**) Scatter plot of mean strain ratio measured by USE and mean Young’s modulus measured by mechanical testing (strain range 10%–20%). Dashed curve indicates a hyperbola fit.

Three spinal cords originally from non-paralyzed rabbits and another three spinal cords originally from paralyzed rabbits were harvested for mechanical testing. From them, a total of 17 samples ($\approx$ 3 mm long) were obtained and transversely loaded during the compression test. T1-weighted MR images and the same compounded with axial normal strain elastograms (spinal canal segmented and normalized with respect to the surrounding soft tissues) acquired from representative transverse planes of a non-paralyzed rabbit and a paralyzed rabbit are presented (Fig. 3A). From each of the same two rabbits, stress–strain curves of three samples are also presented (Fig. 3B). In general, samples from non-paralyzed rabbits demonstrate larger slopes than that of paralyzed rabbits in the stress–strain curve across the entire testing range, whereas USE strains are higher from the paralyzed case than that of the non-paralyzed case, consistently with results in Figs. 1B and 2B. Results from USE and mechanical testing corroborate each other as the strain and Young’s modulus are by nature inversely proportional to each other. The strain ratios and Young’s moduli (the latter of which were obtained from the strain range 10%–20%) are spatially averaged across each rabbit’s laminectomy defect area and plotted (Fig. 3C). The strain ratio-modulus distribution appears to align with a hyperbola (by least-square fit) suggesting inverse proportionality.

### Association between USE and DTI tractography

**Figure 4:**
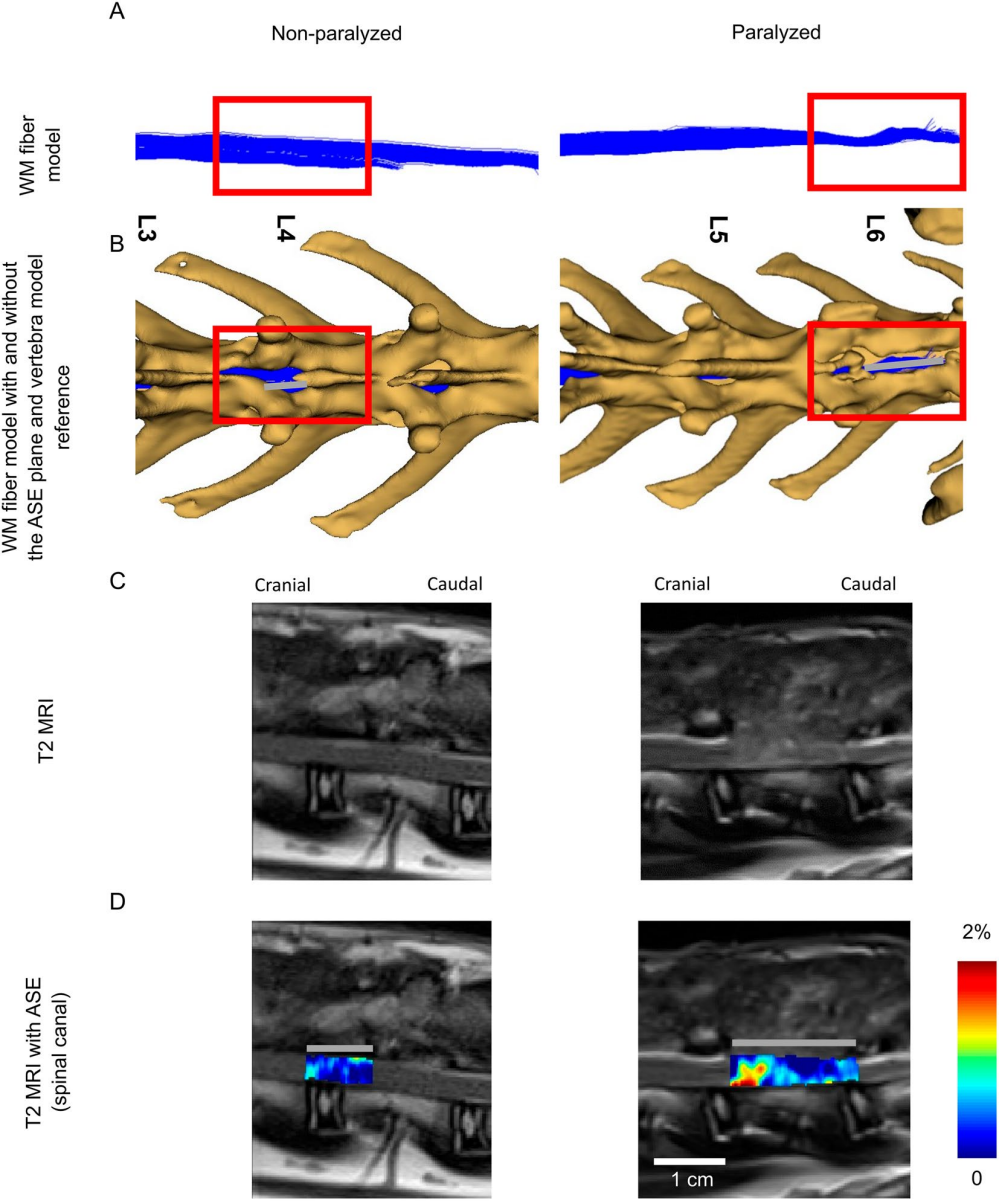
Association between USE and DTI tractography. (**A**) White matter fiber model reconstructed from DTI tractography alone and (**B**) compounded with vertebral bone model reconstructed from CT. (**C**) T2-weighted MR images alone and (**D**) compounded with axial normal strain elastogram (with spinal canal segmented). The left column corresponds to a non-paralyzed rabbit and the right column corresponds to a paralyzed rabbit. *WM* White matter.

Diffusion weighted MR data from one non-paralyzed rabbit and another paralyzed rabbit were processed for white matter fiber reconstruction (Fig. 4A). From each of the same two rabbits, T2-weighted MR images alone and compounded with axial normal strain elastograms (spinal canal segmented) are also presented (Fig. 4C,D). ROIs of USE in relation to the reconstructed white matter fiber tracts are annotated and referenced to the surrounding vertebral bones containing the laminectomy defect area (Fig. 4B,D, gray lines). Tractography of the paralyzed rabbit indicates marked differences in the fiber local orientation compared to that of the non-paralyzed rabbit (Fig. 4A, boxed areas). From the paralyzed rabbit, USE shows pronounced axial normal strains localized within the spinal cord and close to the cranial end of the laminectomy defect in the presented sagittal view, whereas strains in the non-paralyzed rabbit’s spinal cord are approximately uniform across the sagittal view of the laminectomy defect (Fig. 4D). Changes in tractography and USE occur at approximately the same location (Fig. 4A,B,D). In the supplementary information, we report USE and T2-weighted MR imaging results from another two non-paralyzed rabbits (Fig. 5 of the supplementary information) and another two paralyzed rabbits (Fig. 6 of the supplementary information). From the paralyzed rabbits, the strain localization patterns are consistently pronounced. In comparison, the strains show a more uniform spatial distribution from the non-paralyzed rabbits.

## Discussion

In this paper, we present a study to assess uninjured and injured spinal cords in a rabbit animal model, in vivo, using USE. USE measures tissue local strains, which have been proven to vary with pathological changes^29^. Moreover, tissue cells have been found to respond to the stiffness of their microenvironment^53^. In the specific context of spinal cord imaging, an ultrasonic tool to characterize the intrinsic variation of strains caused by SCI pathophysiologic processes could improve SCI detection and assessment. In our study, a surgical laminectomy was performed on all rabbits and SCI was created from selected rabbits by applying additional pressure to the spinal cord. USE was applied after confirmation of paralysis among the rabbits with spinal cord injury and no paralysis among the controls, approximately one week post-surgery for all animals. Significant differences between the paralyzed rabbits and non-paralyzed rabbits were detected using USE. A validation study was conducted using independent histologic analysis and mechanical testing. CT and MRI modalities were also used to corroborate findings from USE. Results obtained from this study demonstrate that USE could assess changes caused by the crush injury in the spinal cord of the given animal model. In the future, markers derived from USE could be used to complement and improve existing SCI diagnosis and prognosis methods.

From histology and mechanical testing, the score of myelomalacia and measurement of Young’s modulus were used to validate USE results. In pathology, myelomalacia refers to the structural disruption and softening of the spinal cord^54, 55^. The pathophysiology of traumatic myelomalacia involves mechanical damage to the spinal cord in its primary stage, which could be followed by further damages in its secondary stage^55^. Myelomalacia could have poor prognosis^54^ with symptoms ranging from loss of motor functions to paralysis of the respiratory system. In this study, the strain ratio derived from USE has a higher value when the underlying malacia histologic score is high (and vice versa) in both the dorsal and ventral sections of the ROI. As a result, disruption in the spinal cord architecture may manifest in elastograms as regions of relatively high strains. Young’s modulus was measured in similar mechanical testing conditions to that for USE experiments to validate spinal cord stiffness assessment. From USE, stiffness is loosely interpreted as the inverse of the measured axial normal strain component, i.e., typically low strains indicate high stiffness. Based on our experimental results, the strain ratio/Young’s modulus scatter plot exhibits a qualitatively inverse proportional pattern, suggesting that spinal cord regions exhibiting low USE strains are mechanically stiffer than those exhibiting higher USE strains.

In addition to transverse imaging planes, selected paramedian planes are also presented to examine the qualitative distribution of the strains across the window of laminectomy. Local variations in the strain spatial distribution may provide unique opportunities for accurate localization of the injury in the spinal cord, which in turn can navigate the imaging tool to dedicated ROIs for more detailed evaluation. From both Fig. 4 in the main paper and Fig. 1 in the supplementary information, it is clear that spatial strain concentrations can create a new local contrast mechanism within the spinal cord in paralyzed rabbits. Localization achieved with USE is also consistent with results from DTI tractography by tracking the portion of white matter tracts deflecting from its principal orientation and referring both DTI and axial normal strain elastogram ROIs to their surrounding vertebra landmarks. With automatic registration techniques available^48^, in the future, elastograms and their co-planar B-mode images may be efficiently used to generate multi-modal US/MRI or US/CT images. This could be especially beneficial for the assessment of challenging cases that typically would require multiple independent diagnostic modalities.

We now discuss several assumptions and limitations of this study. In this study, laminectomy was applied to all rabbits to allow US imaging to access the spinal cord. It should be noted that in the acute period of SCI, a significant number of patients undergo spine surgeries^56^. For example, in the year of 2021, an average of 80.5% patients underwent spinal surgery following SCI, according to the National Spinal Cord Injury Statistical Center (NSCISC) (https://www.nscisc.uab.edu/public_pages/reportsstats). As a result, the laminectomy procedure in our study design is clinically relevant. The same or similar laminar bony window has also been used in other procedures such as circumferential decompression (CD) of patients with thoracic ossification of the posterior longitudinal ligament (T-OPLL)^18^ and monitoring the intraspinal pressure (ISP) of patients after severe traumatic SCI^57^.

The animal model in this study was used to only compare spinal cords with complete SCI and non-injured controls. To create a complete SCI, additional pressure was applied to the spinal cord during laminectomy. Consistent functional lesions were obtained in all animals in the experimental group at the time of imaging, demonstrating the repeatability and reliability of this surgical approach. As a result, this study can serve as the first step toward assessing USE as a clinically relevant tool for SCI diagnosis and prognosis. When mean strain ratios from the paralyzed and non-paralyzed animal groups were compared, we noticed a larger standard deviation from the former animal group, which may be caused by variability of the created complete SCI. For future studies, more controlled approaches can be utilized to obtain reproducible incomplete lesions. Availability of intermediate severities of SCI allows representation of a broader patient population in a clinical setting. It is also particularly relevant for therapeutic intervention of SCI. These opportunities could be explored under more systematical study designs featuring in spatiotemporal assessment of SCI using USE and more dedicated histopathologic validation.

Allocation of animals to each validation test was aimed mainly to provide multi-aspect interpretation of USE imaging results of SCI in a proof-of-concept manner. In a general experimental setting, multiple factors may be considered to guide sample allocation and improve stratification of SCI within each validation test. In this study, as we did not include incomplete SCI, we ensured each test had at least a pair of paralyzed and non-paralyzed animals. This selection criterion was not meant to bring any statistical validity for hypothesis testing, but to obtain standard testing results to interpret USE measurement from a diversity of SCI conditions given the animal model. Figures 2 and 3 in turn confirmed the cases we selected had distinct strain ratios. These results can lay the foundation for more quantitative studies in the future to correlate clinically relevant SCI severity indicators and various USE parameters.

Among the various strain elastograms that can be computed for USE, only axial normal strain elastograms were considered in this study. Axial normal strain elastography is the method of choice in clinical elastography studies^58, 59^. In noisy imaging environments such as those of an operating room and with complex anatomy typical of spine tissues, a linear array transducer can conveniently generate axial normal strain elastograms of premium image quality in real time and without using contrast agents or stringent experimental conditions. However, in the future, the niche of USE in SCI applications can be greatly extended by improving estimation of other strain components from USE. For example, quality lateral strain elastograms may be combined with axial normal strain elastograms to measure tissue compressibility^50^, whereas availability of volumetric strains from USE may allow derivation of fluid transport parameters of strong clinical relevance, such as fluid pressure, solid stress and vascular permeability^44, 60–64^. Coupled with distinct 3-D spatial organization patterns of the spinal cord, these parameters may further reveal the underlying mechanical and structural properties of spinal cords under normal conditions and with injury. For example, anisotropy has been known as a common attribute of most biological tissues including the CNS tissue^65^. In the field of tissue engineering, anisotropic constitutive models are widely used for an accurate formulation of the desired biomaterial mechanical behavior^66–68^. Therefore, these parameters could be used as sensitive imaging markers to assess other pathophysiological processes of SCI, such as vacuolation, hemorrhage and neuronal degeneration.

## Conclusions

In this study, we investigated the use of USE to assess SCI in a rabbit animal model with non-paralysis and paralysis neurological statuses. The results demonstrate that 1) USE can assess changes in the spinal cord related to the injury from the presented animal model and 2) USE may be an important quantitative tool for SCI diagnosis and prognosis.

### Supplementary Information

Supplementary Information.

## Data Availability

The datasets generated and/or analyzed during the current study are available from the corresponding author on reasonable request.
