# Supplementary material for: Assessment of spinal cord injury using ultrasound elastography in a rabbit model in vivo
Source: Sci Rep. 2023 Sep 15;13:15323. doi: 10.1038/s41598-023-41172-8 (PMC10504274; doi:10.1038/s41598-023-41172-8)
Supplement: Supplementary file 1 — Supplementary Information. [file 41598_2023_41172_MOESM1_ESM.docx]

# **Assessment of spinal cord injury using ultrasound elastography in a rabbit model *in vivo***

## **Supplementary information**


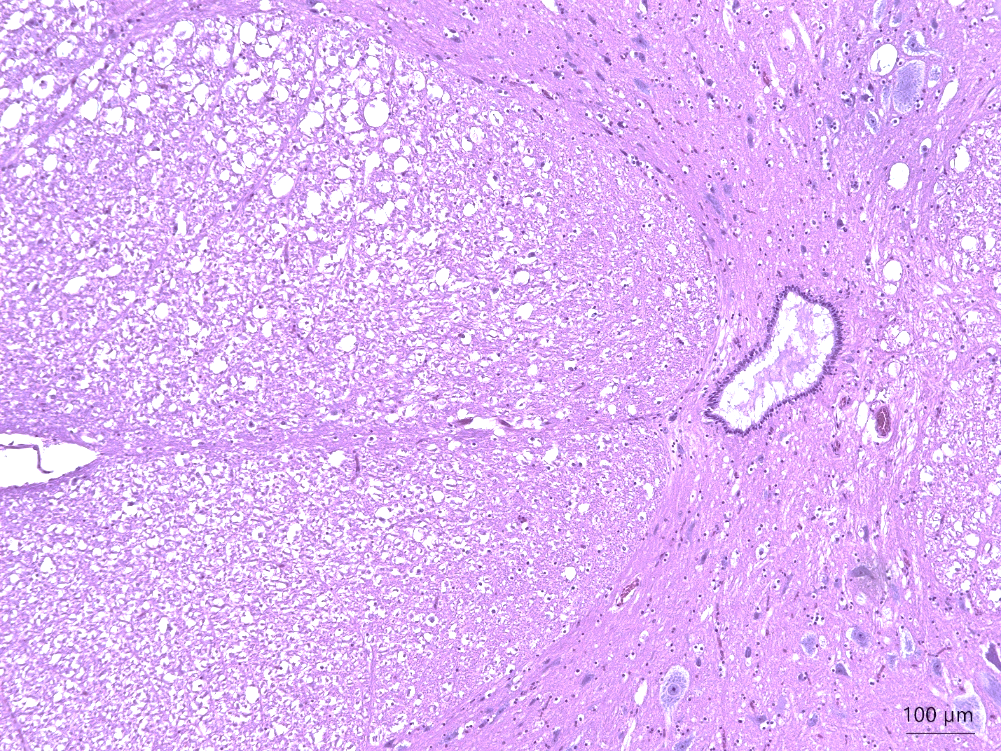


Fig. 1: Dorsal region of the non-paralyzed animal’s histological section in Fig. 2 of the manuscript.


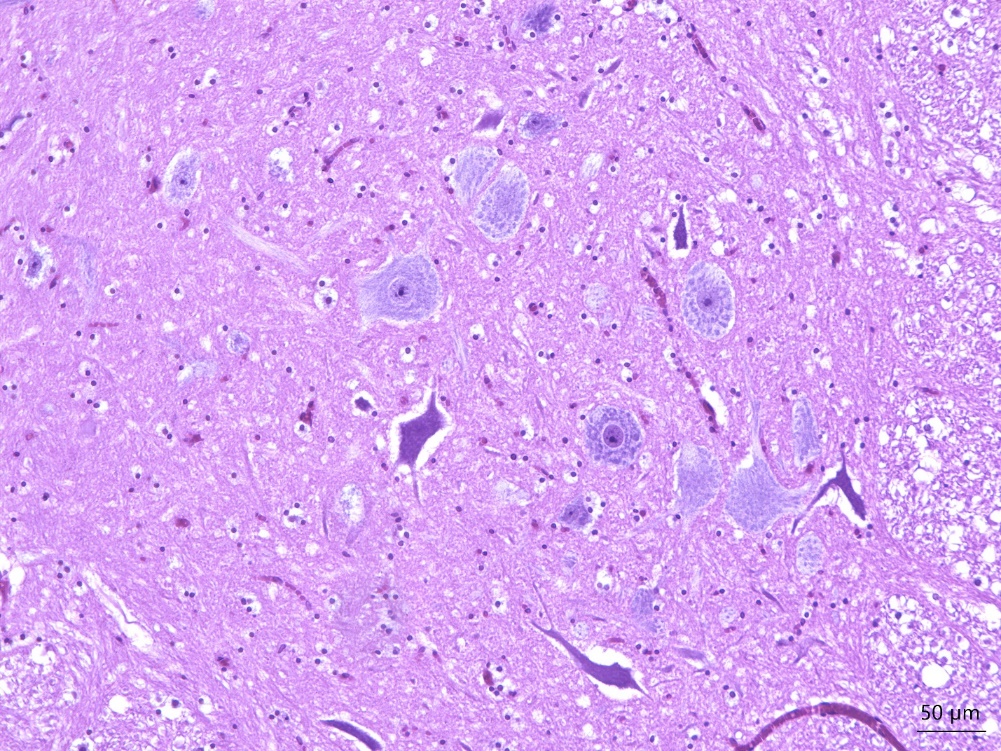


Fig. 2: Gray matter interior region of the non-paralyzed animal’s histological section in Fig. 2 of the manuscript.


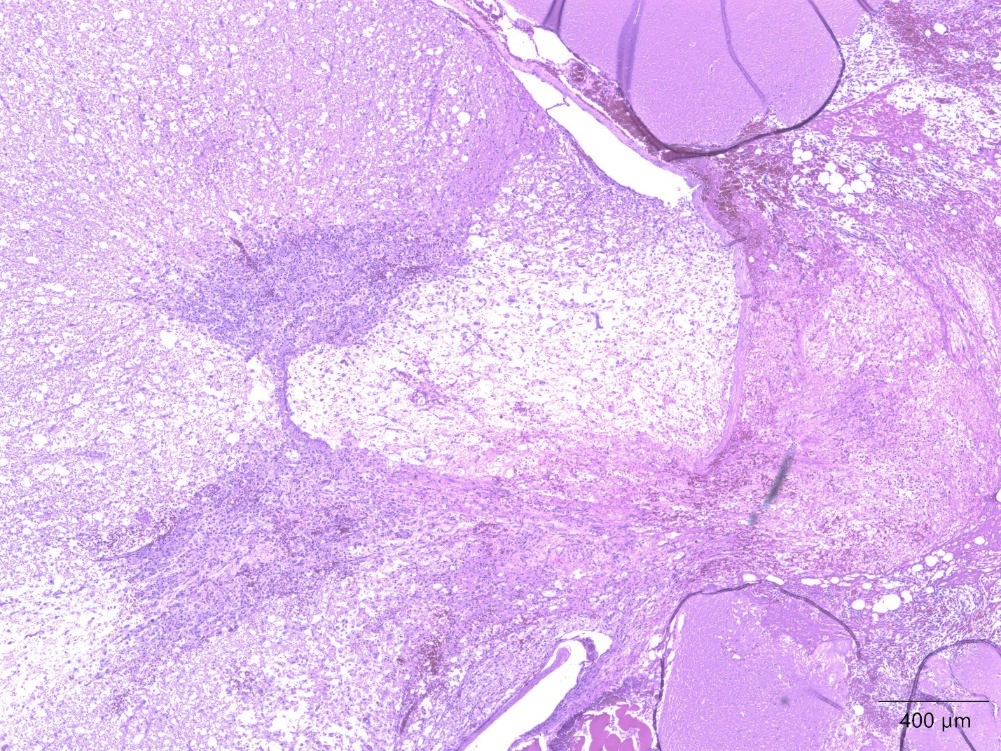


Fig. 3: Dorsal region of the paralyzed animal’s histological section in Fig. 2 of the manuscript.


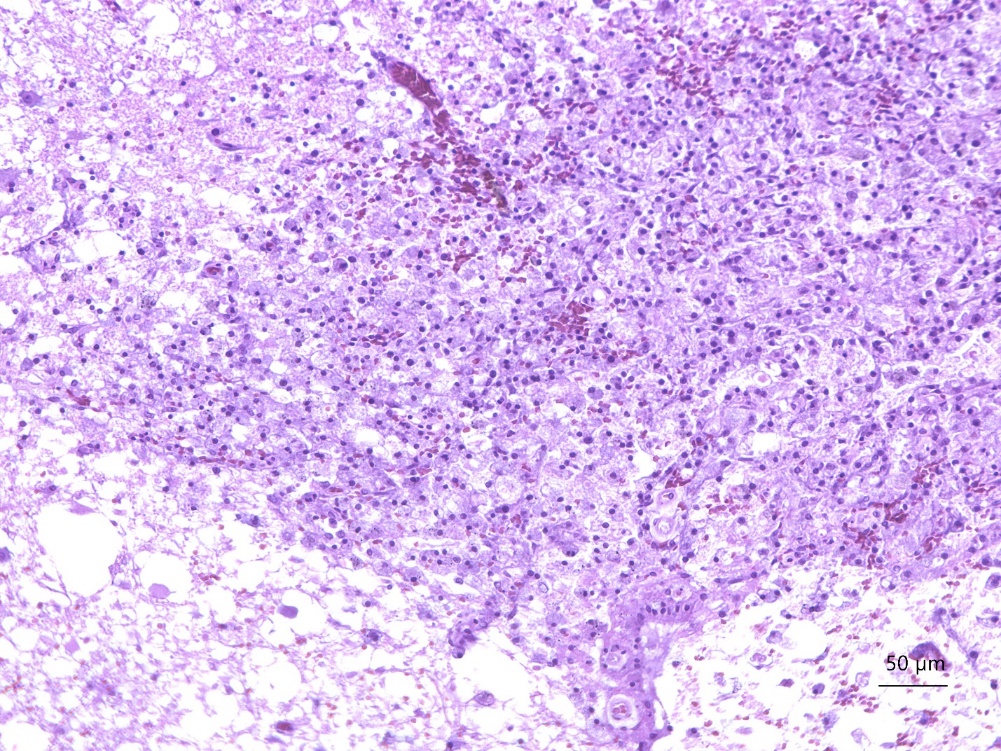


Fig. 4: Gray matter interior region of the paralyzed animal’s histological section in Fig. 2 of the manuscript.


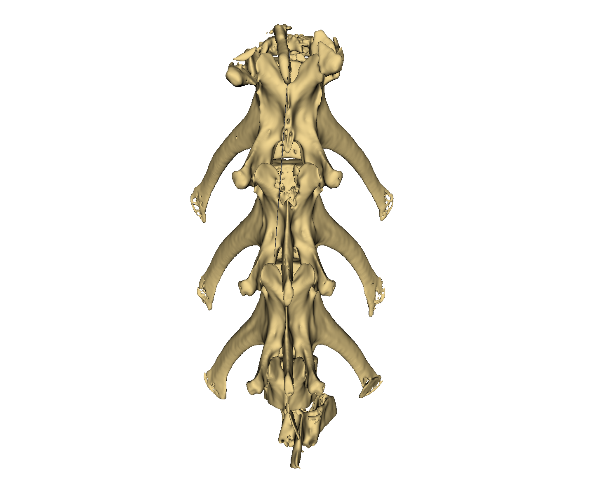


**L5**

**L6**


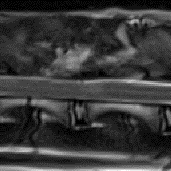

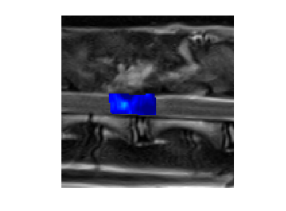


**T2 MRI no compounding**

**T2 MRI compounded with USE**

0 (mm)

-18.9

18.9

Caudal

Cranial

0 (mm)

-18.9

18.9

Caudal

Cranial

0 (mm)

40

40

20

20

0 (mm)


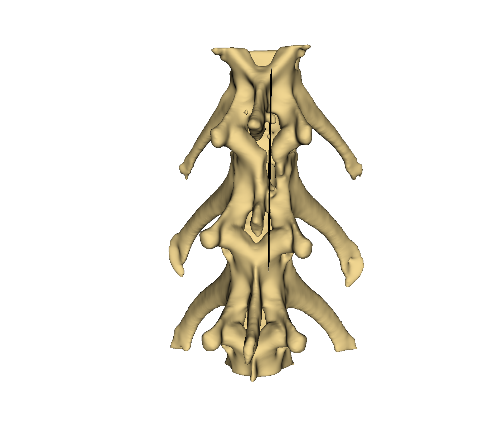


**L5**

**L6**


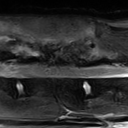

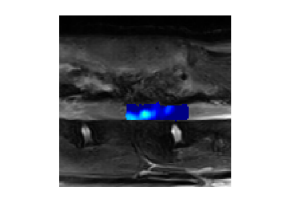


Caudal

Cranial

0 (mm)

-18.9

18.9

Caudal

Cranial

0 (mm)

-18.9

18.9

0 (mm)

40

0 (mm)

40

20

20

**T2 MRI no compounding**

**T2 MRI compounded with USE**

Fig. 5: Multimodal imaging results obtained in paramedian planes from 2 non-paralyzed rabbits. For each rabbit, the three columns from left to right show the imaging plane referenced to the vertebral bone model reconstructed from CT, the T2-weighted MR image alone and compounded with axial normal strain elastogram (spinal canal segmented). The contrast in each image was adjusted to show the strain distribution along the spinal cord.


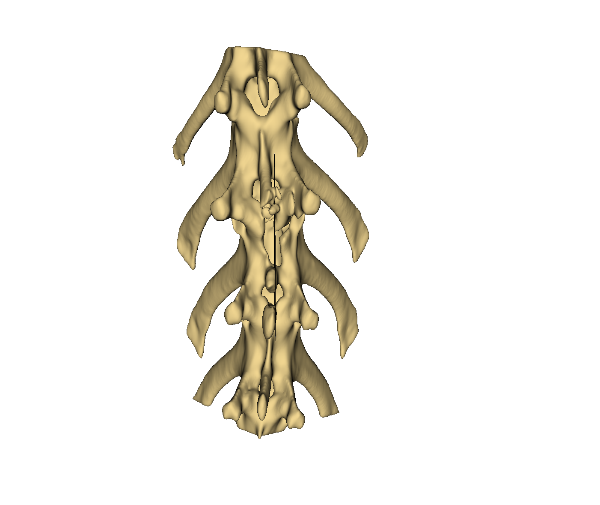


**L4**

**L5**


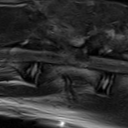

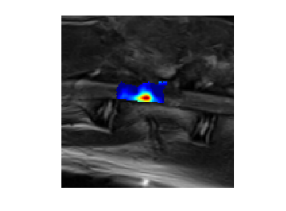


Caudal

Cranial

0 (mm)

-18.9

18.9

Caudal

Cranial

0 (mm)

-18.9

18.9

0 (mm)

40

20

0 (mm)

40

20


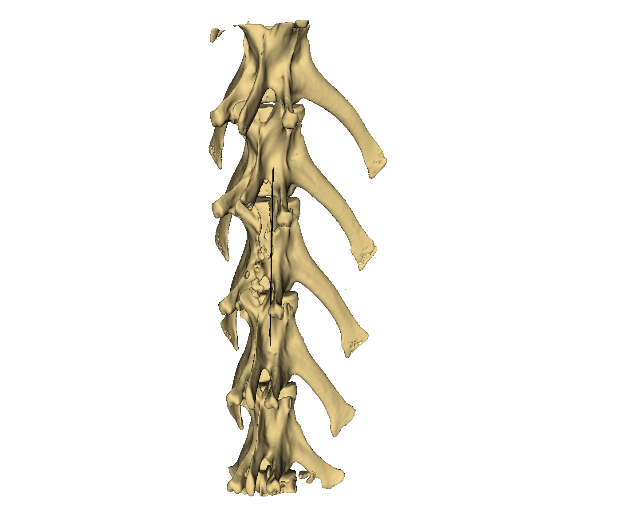


**L4**

**L5**


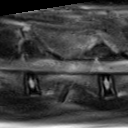

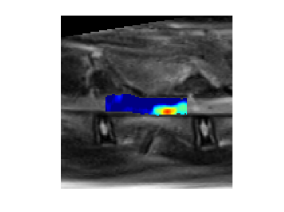


Caudal

Cranial

0 (mm)

-18.9

18.9

Caudal

Cranial

0 (mm)

-18.9

18.9

0 (mm)

40

20

0 (mm)

40

20

**T2 MRI no compounding**

**T2 MRI compounded with USE**

**T2 MRI no compounding**

**T2 MRI compounded with USE**

Fig. 6: Multimodal imaging results obtained in paramedian planes from 2 paralyzed rabbits. For each rabbit, the three columns from left to right show the imaging plane referenced to the vertebral bone model reconstructed from CT, the T2-weighted MR image alone and compounded with axial normal strain elastogram (spinal canal segmented). The contrast in each image was adjusted to show the strain distribution along the spinal cord.
